# Infective endocarditis in an adult with undiagnosed tetralogy of Fallot: a case report of a rare presentation

**DOI:** 10.1186/s43044-024-00582-3

**Published:** 2024-11-13

**Authors:** Mahmoud Gomaa, Ahmed Shaban, Hassan El-Shirbiny, Anas Elgenidy

**Affiliations:** 1 Department of Cardiovascular Medicine, Kafrelsheikh University Hospital, Kafrelsheikh, 33511 Egypt; 2 Faculty of Medicine, Kafrelsheikh University, Kafrelsheikh, Egypt; 3 Faculty of Medicine, Cairo University, Cairo, Egypt; 4 Karl-Jaspers-Klinik, Zwischenahn, Germany

**Keywords:** Tetralogy of fallot, Infective endocarditis, Congenital heart disease

## Abstract

**Background:**

Unrepaired tetralogy of Fallot (TOF) is uncommonly diagnosed in adulthood and only 3% of patients survive to reach the age of 40 without surgical repair. If unrepaired, these patients are at risk for infective endocarditis (IE).

**Case presentation:**

In this report, we present a case of a middle-aged, previously healthy female whose only complaint was unexplained fever. Echocardiography led to the discovery of undiagnosed TOF complicated with IE with a vegetation on the right ventricular (RV) side of the ventricular septal defect (VSD) which was appropriately managed with antibiotics.

**Conclusions:**

In rare cases of acyanotic TOF where there is a lesser degree of right ventricular outflow tract obstruction (RVOTO), patients may survive into adulthood and can be asymptomatic till becoming initially presented with complications such as infective endocarditis.

## Background

Tetralogy of Fallot (TOF) is the most common cyanotic congenital heart disease (CHD), accounting for 7–10% of all CHD cases. It involves four main defects: a ventricular septal defect (VSD), an overriding aorta, right ventricular outflow tract obstruction (RVOTO), and right ventricular hypertrophy (RVH). Only 3% of patients with unrepaired TOF survive to age 40, making it rare in adults without surgical correction [1].

Survival without surgery can occur when there is a balanced bidirectional shunt or a left-to-right shunt, making the condition acyanotic. This is often associated with compensatory adaptations like developing collateral circulation or systemic hypertension [2]. These patients often experience symptoms similar to those with large VSDs, such as fatigue and shortness of breath. Death typically results from chronic congestive heart failure due to prolonged right ventricular pressure overload, arrhythmias, or, rarely, infective endocarditis.

Structural abnormalities in unrepaired TOF carry a risk for infective endocarditis, which increases with age [3]. Infected vegetations can form on the right ventricular endocardium, pulmonic valve, RVOT, or around the VSD edges.

In this report, we present a case of previously undiagnosed TOF that presented with fever, and investigations revealed infective endocarditis. This highlights the importance of considering such complications in patients with fever of unknown origin, as they may have an underlying undiagnosed congenital heart disease [4].

## Case presentation

**Fig. 1:**
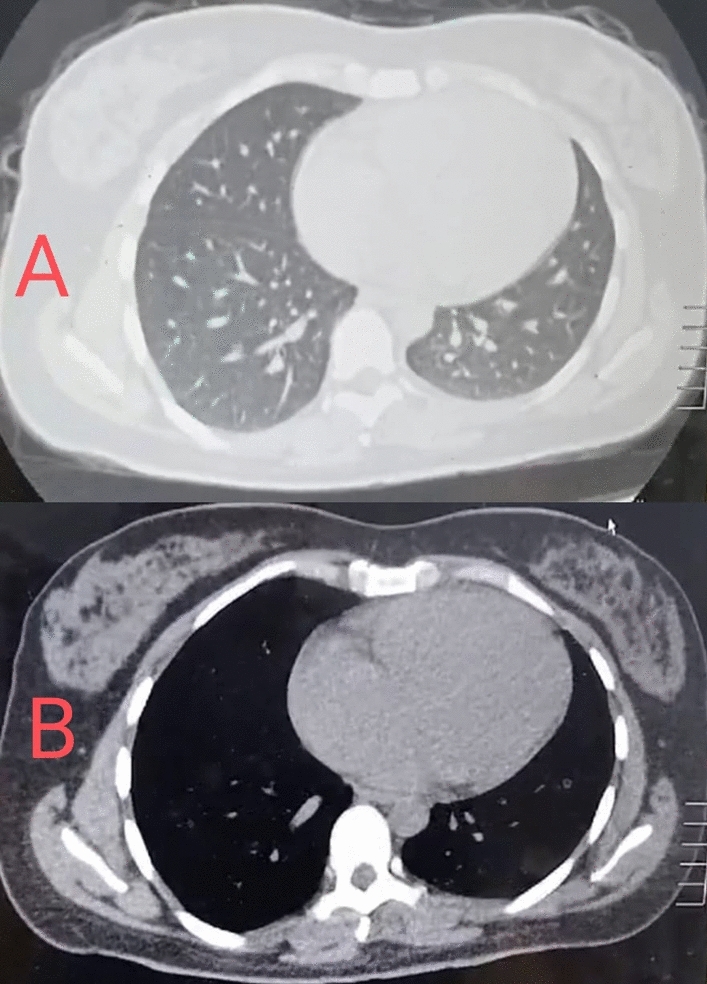
Axial CT chest: (**A**) Lung window of the CT chest with no signs of pneumonic or interstitial infiltration, (**B**) Mediastinal window suggestive of right side dilatation.

Our case is a 35-year-old single female with no significant past medical or surgical history. For the past 6 weeks before presentation, she had been complaining of fatigue, exertional dyspnea, recent weight loss and fever that responded partially to antipyretics, but never disappeared more than 3 days. Initially, she went to a pulmonologist who reported no signs of chest infection, but he noticed a significant murmur on auscultation and she had a CT chest in which the right side of the heart seemed dilated (Fig. 1).

Consequently, she was referred to us, and upon questioning, she stated that she her dyspnea had been present for the past 2 years, but her current main complaint was the fever that increased her dyspnea and affected her daily activities and farming work.

**Fig. 2:**
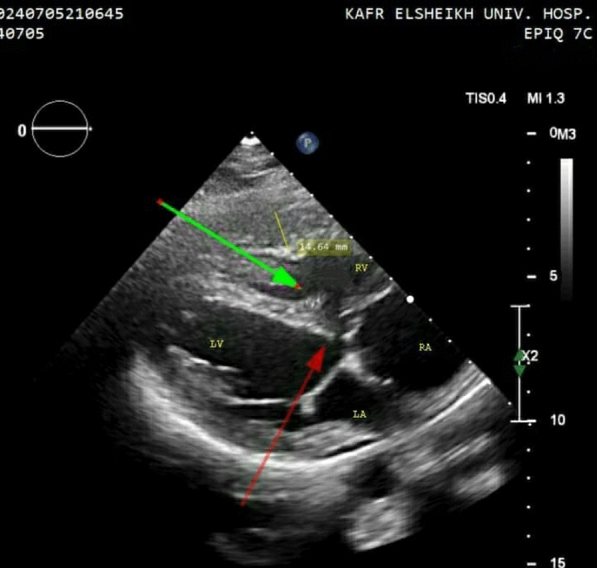
PSLA view of TTE showing features of TOF (RVH, overriding aorta and VSD (red arrow)) and features suggesting IE (an echodense, irregular mass (vegetation) on the RV side of the VSD (green arrow))

**Fig. 3:**
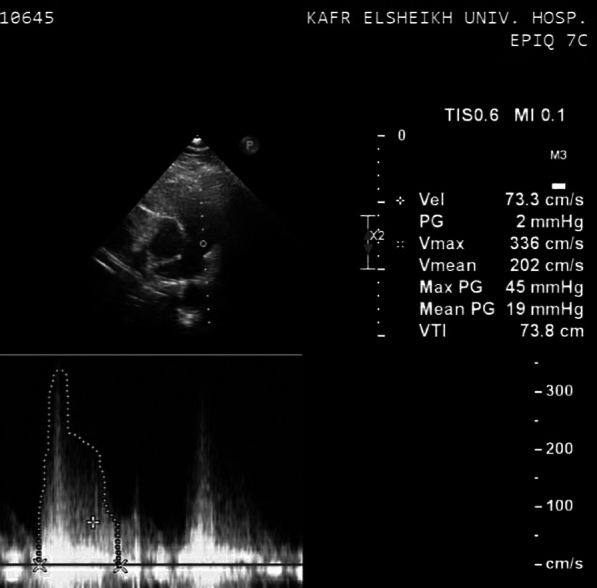
PSSA view on TTE showing moderate degree of RVOTO with a maximum gradient of 45 mmHg at the RVOT

Examination revealed normal peripheral oxygen saturation (Spo2 94%) with no cyanosis or signs of congestive heart failure. However, cardiac auscultation revealed a single second heart sound (S2) and a harsh systolic murmur at the left upper sternal border. Her ECG showed right axis deviation, 0.5 mm ST segment depression in leads V1 and V2 and deep S wave in lead V6. Transthoracic echocardiography was done which showed typical features of tetralogy of Fallot (TOF): a VSD of 5 mm in diameter with bidirectional flow (mainly left to right) with an echodense, irregular, mobile mass 10.2 mm long and 4.27 mm wide on the edge of the VSD at the right ventricular outflow tract (RVOT) (Fig. 2), an overriding aorta over the septum ≤ 50%, increased thickness of the RV wall (14.6 mm), and moderate degree of RVOT obstruction (maximum gradient of 45 mmHg) (Fig. 3).

The patient was admitted to the cardiac care unit with a provisional diagnosis of infective endocarditis. She was vitally stable and her temperature was 38.9 degrees. Three blood cultures from 3 different sites were withdrawn with a time interval of one hour, and she was started on empirical antibiotic therapy of Ampicillin/Sulbutam (12 gm/day) and Gentamycin (3 mg/kg/day) that was continued for the next 2 weeks.

Laboratory findings of the admission day were non-relevant except for an elevated total leucocytic count of 15.7*10^3^/ml with neutrophilic predominance and an elevated C-reactive protein level of 96. On the next day, transesophageal echocardiography (TEE) was done, and based on Duke’s criteria, the patient had 2 major and 2 minor criteria and was, therefore, diagnosed as a case of confirmed IE [5]. Therefore, therapy was continued. On the fifth day, her temperature dropped to 37.3 degrees and she reported improvement in her general condition. Culture results appeared one week after admission and were positive for Viridans Streptococci which, fortunately, was sensitive to the antibiotics she was receiving.

**Fig. 4:**
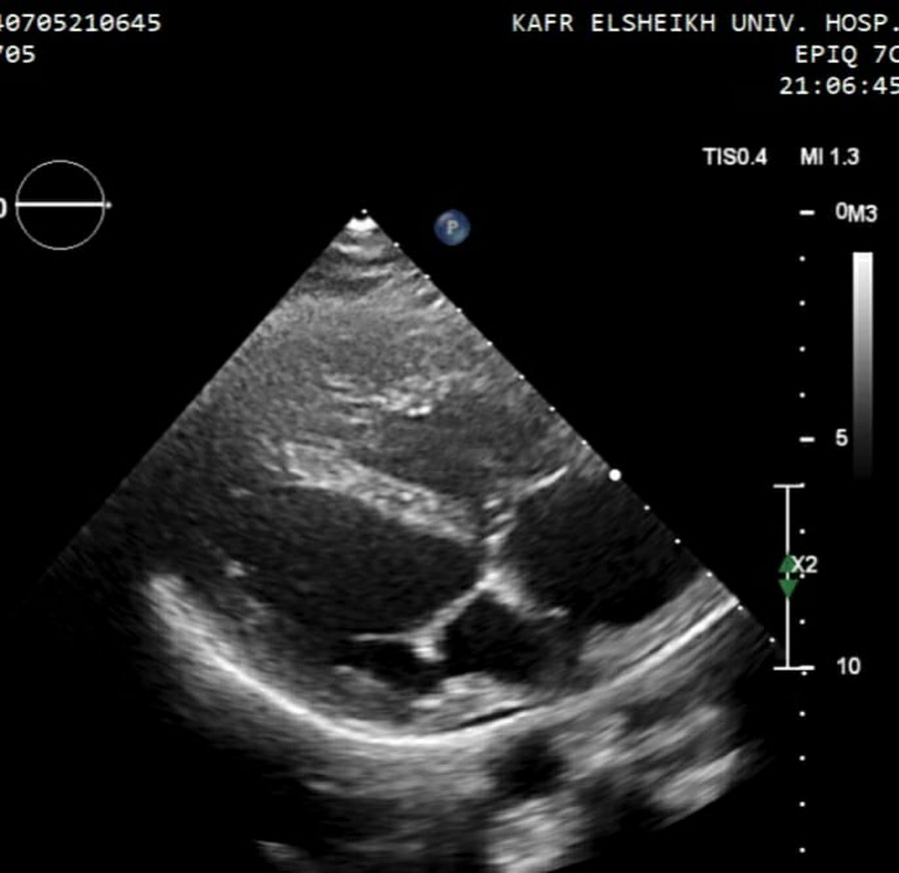
PSLA view in TTE after treatment showing resolution of the vegetation after 2 weeks of antibiotic therapy

Two weeks after admission, the patient labs came with a total leucocytic count of 9.6*10^3^/ml and a negative C-reactive protein. TTE was repeated and the vegetation had been resolved [Fig. 4]. Subsequently, she was discharged after 15 days from the date of admission.

## Discussion

This case report highlights a rare presentation of Tetralogy of Fallot (TOF) with a potentially fatal complication. A middle-aged patient with no significant medical history sought medical advice for persistent fever. After six weeks without identifying an infection source, a cardiologist referral was made to investigate possible infective endocarditis. Echocardiography revealed TOF features and a vegetation on the VSD edge. Blood cultures confirmed Streptococci, diagnosing infective endocarditis.

Tetralogy of Fallot (TOF) is usually diagnosed at birth due to cyanosis. However, in rare cases, it can go unnoticed until adulthood if there is a balance between the RVOTO and VSD, preventing cyanosis [6]. Our patient, first presenting at the age of 35 with fatigue and unexplained fever, was found to have infective endocarditis due to previously undiagnosed TOF.

Most adults with unrepaired TOF are aware of their condition and have previously refused surgery. For example, Subhawong et al. reported an 87-year-old patient with uncorrected TOF who presented with an acute lateral basal ganglia infarct. Her medical records showed that she had been followed by a cardiologist and had declined surgical repair for her CHD [7].

There are a few case reports of adults with accidentally discovered TOF. For example, Hakim et al. reported a 56-year-old patient whose chest CT angiography, done to rule out pulmonary embolism, revealed left pulmonary artery and hypoplastic main and right pulmonary arteries. Further echocardiography confirmed the diagnosis of TOF with a 16 mm VSD, a bidirectional VSD shunt, hypertrophied right ventricle with borderline systolic function and an aorta overriding the IVS by 50%. The patient was mostly asymptomatic all his life [8]. Similarly, Alkashkari et al. described a 29 year-old male diagnosed with TOF after investigating unexplained dyspnea [9].

In CHDs, several factors increase the risk of IE, most importantly due to turbulent blood flow from structural abnormalities [10]. This is a significant condition for both pediatric and adult CHD patients. However, adults with CHDs are more vulnerable to IE due to complex cardiac conditions and higher comorbidities. The incidence of IE in adults with CHD is 11 per 100,000 person-years, three times higher than in children with CHD. Additionally, the inpatient mortality for IE can reach up to 5.0–6.7% in children compared to 8.8–15% in adults [11].

IE is a serious complication especially in patients with multiple comorbidities and often leads to prolonged hospitalization. A notable study showed that 3.5% of TOF patients were hospitalized for IE compared to less than 0.8% of controls [12].

In the current era, surgical repair of TOF is still recommended even in asymptomatic patients with accidental or late diagnosis, especially with an acceptable surgical risk and lower mortality rate on long term [13]. However, individualized risk assessment should be done before taking the surgery decision. Yang et al. presented a report of a case diagnosed with TOF at age 31 but did not seek further medical care. At age 66, he was reassessed for proteinuria and found to have mild cyanosis, clubbing, and various heart abnormalities. He was managed medically due to his age and asymptomatic status until he developed palpitations and dyspnea at age 69, leading to worsening condition and recurrent hospitalization until he died at age 73 [14].

Our patient was a candidate for surgical repair of TOF, and she was educated about her condition and the possible complications if left untreated, but she refused any surgical intervention. However, she was pleased with the medical care she got and that her fever had resolved and she could resume her farming work. She was advised to follow up regularly every 6 months, and to seek medical care if she ever developed unusual symptoms such as fever, dyspnea, fatigue or weight loss.

## Conclusion

This case provides a rare insight into a late, potentially fatal outcome of uncorrected TOF that was not previously diagnosed in a middle-aged, previously healthy patient. Infective endocarditis should be taken into consideration in patients with unexplained fever even without obvious risk factors because of the possibility of being associated with asymptomatic CHD such as TOF.

## Data Availability

The data of this case report includes the echocardiography film and all other patient’s data that we a had a consent to publish. These data are available from the corresponding author on reasonable request.

## Abbreviations

TOF: Tetralogy of fallot
CHD: Congenital heart disease
IE: Infective endocarditis
RVOT: Right ventricular outflow tract
RVOTO: Right ventricular outflow tract obstruction
VSD: Ventricular septal defect
TTE: Transthoracic echocardiography
TEE: Transesophageal echocardiography
PSLA: Parasternal long axis
PSSA: Parasternal short axis

## References

[1] Bhattarai P, Karki M, Purewal JK, Devarakonda PK (2023) Unrepaired tetralogy of fallot: a tale of delayed presentation and limited access to care. J Cardiol 30(2):123–135. 10.1234/jcard.2023.4567

[2] Chandrasekaran B, Wilde P, McCrea WA (2007) Tetralogy of fallot in a 78-year-old man. N Engl J Med 357(11):1160–1161. 10.1056/NEJMc063349 17855682 10.1056/NEJMc063349

[3] Freed MD (1993) Infective endocarditis in the adult with congenital heart disease. Cardiol Clin 11(4):589–602 8252561

[4] Carvajal V, Reyes FB, Gonzalez D, Schwartz M, Whiltlow A, Alegria JR (2024) Endocarditis in adult congenital heart disease patients: prevention, recognition, and management. Curr Cardiol Rep 26(9):1031–1045. 10.1007/s11886-024-02103-9 39212775 10.1007/s11886-024-02103-9 PMC11379749

[5] Fowler VG Jr, Durack DT, Selton-Suty C, Athan E, Bayer AS, Chamis AL et al (2023) The 2023 duke-international society for cardiovascular infectious diseases criteria for infective endocarditis: updating the modified duke criteria. Clin Infect Dis. 10.1093/cid/ciae040 37138445 10.1093/cid/ciad271 PMC10681650

[6] Johns Hopkins Medicine. “Tetralogy of Fallot in Adults.” Johns Hopkins Medicine Health Library. Accessed [https://www.hopkinsmedicine.org/health/conditions-and-diseases/continuing-tetralogy-of-fallot-treatment-into-adulthood]

[7] Subhawong TK, Teytelboym O (2009) Survival to the age of 87 years in a woman with unoperated tetralogy of Fallot. J Radiol Case Rep. 3(8):14–7. 10.3941/jrcr.v3i8.264 22470677 10.3941/jrcr.v3i8.264 PMC3303331

[8] Hakim K, Benothman R, Mekki N et al (2023) Paucisymptomatic Tetralogy of Fallot diagnosed in a 56-year-old patient: a case report. Egypt Heart J 75:42. 10.1186/s43044-023-00372-3 37233917 10.1186/s43044-023-00372-3 PMC10219914

[9] Alkashkari W, Al-Husayni F, Almaqati A, AlRahimi J, Albugami S (2020) An adult patient with a tetralogy of fallot case. Cureus 12(11):e11658. 10.7759/cureus.11658.PMID:33391897;PMCID:PMC7769492 33391897 10.7759/cureus.11658 PMC7769492

[10] Ferrieri P, Gewitz MH, Gerber MA, Newburger JW, Dajani AS, Shulman ST, Wilson W, Bolger AF, Bayer A, Levison ME, Pallasch TJ, Gage TW, Taubert KA (2002) Unique features of infective endocarditis in childhood. Pediatrics 109(5):931–943. 10.1542/peds.109.5.931. (**PMID: 11986458**) 11986458 10.1542/peds.109.5.931

[11] Mulder BJ (2013) Endocarditis in congenital heart disease: who is at highest risk? Circulation 128(13):1396–1397. 10.1161/CIRCULATIONAHA.113.005220 24060941 10.1161/CIRCULATIONAHA.113.005220

[12] Havers-Borgersen E, Butt JH, Smerup M, Gislason GH, Torp-Pedersen C, Gröning M, Schmidt MR, Søndergaard L, Køber L, Fosbøl EL (2021) Incidence of infective endocarditis among patients with tetralogy of fallot. J Am Heart Assoc. 10(22):e022445. 10.1161/JAHA.121.022445 34730003 10.1161/JAHA.121.022445 PMC8751965

[13] van der Ven JPG, van den Bosch E, Bogers AJ, Helbing WA (2019) Current outcomes and treatment of tetralogy of Fallot. F1000Research 8:1530. 10.12688/f1000research.17174.1 10.12688/f1000research.17174.1 PMC6719677 31508203

[14] Yang X, Freeman LJ, Ross C (2005) Unoperated tetralogy of Fallot: case report of a natural survivor who died in his 73rd year; is it ever too late to operate? Postgrad Med J 81(952):133–134. 10.1136/pgmj.2004.020172 15701749 10.1136/pgmj.2004.020172 PMC1743212

